# PPM1H loss alters intrasynaptic alpha-synuclein localization in a LRRK2-dependent manner

**DOI:** 10.1186/s13041-026-01352-9

**Published:** 2026-09-29

**Authors:** Lennart Evers, Jessica M. Grein, C. Alexander Boecker

**Affiliations:** Department of Neurology, University Medical Center Goettingen, 37077 Goettingen, Germany

**Keywords:** LRRK2, PPM1H, RAB GTPases, Alpha-synuclein, Presynapse, Synapse

## Abstract

Mutations in the LRRK2 gene are a major genetic cause of Parkinson’s disease (PD) and induce hyperphosphorylation of RAB GTPases through increased LRRK2 kinase activity. The phosphatase PPM1H counteracts this activity by selectively dephosphorylating LRRK2-phosphorylated RAB proteins. Here, we used primary *PPM1H* knockout neurons to investigate how loss of PPM1H and the resulting hyperphosphorylation of RAB proteins affect the distribution of alpha-synuclein (aSyn), a key protein in PD pathology, at presynaptic terminals. We found that PPM1H deficiency significantly increased the distance between aSyn and the presynaptic marker Bassoon, indicating displacement of aSyn away from the presynapse. Pharmacological LRRK2 kinase inhibition rescued this phenotype, restoring intrasynaptic aSyn positioning to wild-type values. These findings show that loss of PPM1H alters aSyn localization in a LRRK2 kinase activity-dependent manner, suggesting that elevated levels of LRRK2-phosphorylated RAB proteins disrupt the nanoscale organization of aSyn at the presynapse.

**Supplementary Information:**

The online version contains supplementary material available at https://doi.org/10.1186/s13041-026-01352-9.

## Main text

Parkinson’s disease (PD) is a common neurodegenerative disorder characterized by progressive neuronal loss and the aggregation of misfolded alpha-synuclein (aSyn) into Lewy bodies and neurites [1]. Although the precise physiological function of aSyn remains incompletely understood, it is enriched at presynaptic terminals, where it has been implicated in regulating neurotransmitter release [2, 3].

Mutations in the leucine-rich repeat kinase 2 (*LRRK2*) gene are a major genetic cause of familial and sporadic PD. Pathogenic mutations increase LRRK2 kinase activity and induce hyperphosphorylation of a subset of RAB GTPases, key regulators of intracellular trafficking [4–6]. Protein phosphatase 1 H (PPM1H) counteracts LRRK2 activity by selectively dephosphorylating RAB proteins targeted by LRRK2 [7]. Both expression of hyperactive LRRK2 and loss of PPM1H exacerbate aSyn aggregation in neurons exposed to aSyn preformed fibrils (PFFs), implicating RAB hyperphosphorylation in aSyn pathology [8–11]. These findings prompted us to investigate whether LRRK2-mediated RAB hyperphosphorylation also affects the abundance and subcellular localization of endogenous aSyn in neurons. Previous work by Brzozowski et al. demonstrated that pharmacological inhibition of LRRK2 kinase in wild-type (WT) neurons alters the positioning of aSyn within presynaptic terminals [12]. However, it remains unclear whether this effect is mediated by reduced levels of LRRK2-phosphorylated RAB proteins, and conversely, how increased phospho-RAB levels, as seen in the context of LRRK2 hyperactivity or PPM1H loss, affect aSyn positioning at the synapse.

**Fig. 1:**
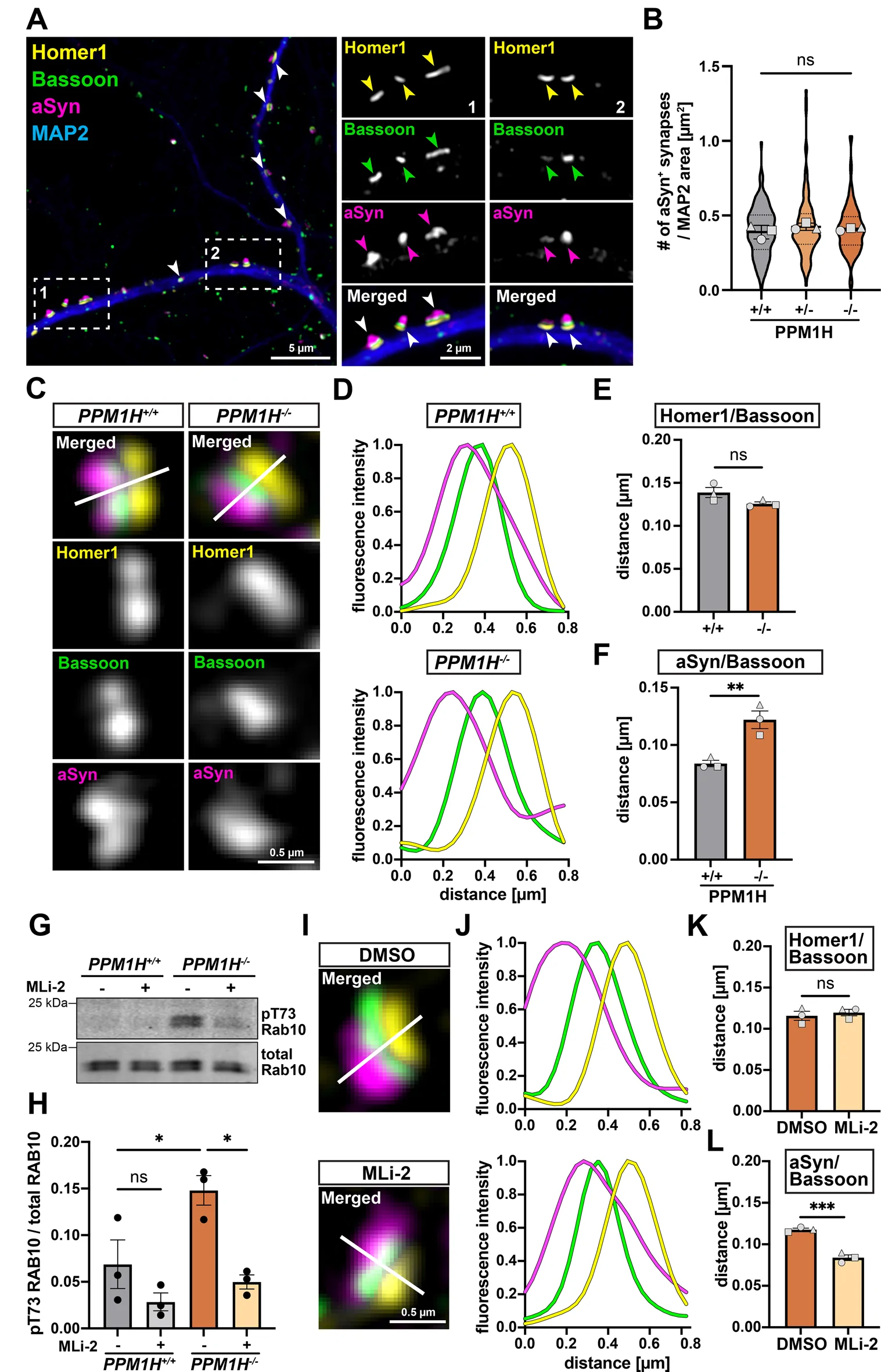
PPM1H deficiency alters intrasynaptic localization of alpha-synuclein. (**A**) DIV21 mouse cortical neurons stained for the postsynaptic marker Homer1 (yellow), the presynaptic marker bassoon (green), aSyn (magenta), and the somatodendritic marker MAP2 (blue). Arrowheads highlight examples of aSyn^+^ synapses. (**B**) Number of aSyn^+^ synapses per µm^2^ MAP2 area in *PPM1H*^*+/+*^*, PPM1H*^*+/-*^, and *PPM1H*^*-/-*^ neurons (*n* = 111–114 fields of view from 3 independent experiments; ns, not significant, *p* > 0.4884; mixed effects model analysis). (**C**) Representative synapses of DIV21 *PPM1H*^*+/+*^ and *PPM1H*^*-/-*^ neurons. (**D**) Line scans of images shown in (**C**) depicting intrasynaptic localization of aSyn. (**E**) Distance between aSyn and Bassoon fluorescence intensity peaks in *PPM1H*^*+/+*^ and *PPM1H*^*-/-*^ neurons (*n* = 72–73 synapses from 3 independent experiments; ** *p* = 0.0047; mixed effects model analysis). (**F**) Distance between Homer1 and Bassoon fluorescence intensity peaks in *PPM1H*^*+/+*^ and *PPM1H*^*-/-*^ neurons (*n* = 72–73 synapses from 3 independent experiments; ns, not significant, *p* = 0.2296; mixed effects model analysis). (**G-H**) Western blot and pT73 RAB10 quantification normalized to total RAB10 in DIV21 *PPM1H*^*+/+*^ and *PPM1H*^*-/-*^ neurons treated with DMSO or 300 nM MLi-2 until cell lysis on DIV21 (mean ± SEM; *n* = 3 biological replicates; ns, not significant; * *p* < 0.0374; one-way ANOVA with Tukey’s multiple comparisons test). (**I**) Representative synapses of *PPM1H*^*-/-*^ neurons treated with DMSO or MLi-2. (**J**) Line scans of images shown in (**I**) depicting intrasynaptic localization of aSyn. (**K**) Distance between aSyn and Bassoon fluorescence intensity peaks in *PPM1H*^*-/-*^ neurons treated with DMSO or MLi-2 (*n* = 72 synapses from 3 independent experiments; *** *p* = 0.0007; mixed effects model analysis). (**L**) Distance between Homer1 and Bassoon fluorescence intensity peaks in *PPM1H*^*-/-*^ neurons treated with DMSO or MLi-2 (*n* = 72 synapses from 3 independent experiments; ns, not significant, *p* = 0.7286; mixed effects model analysis). For panels B, E, F, K, and L, scatter plot points indicate the means of independent experiments, and error bars show mean ± SEM of these points

To address this question, we investigated how elevated phospho-RAB levels resulting from loss of PPM1H affect the abundance and positioning of presynaptic aSyn. Primary cortical neurons from littermate *PPM1H*^+/+^, *PPM1H*^+/-^, and *PPM1H*^-/-^ mouse embryos were fixed at day in vitro (DIV) 21 and immunostained for aSyn, the presynaptic marker Bassoon, the postsynaptic marker Homer1, and the somatodendritic marker MAP2 (Fig. 1A). Synapses were identified based on the apposition of pre- and postsynaptic markers. Quantification showed no significant difference in the fraction of aSyn-positive synapses across genotypes (Fig. 1B), suggesting that loss of PPM1H does not cause overt alterations in the presynaptic localization of aSyn. Our previous work showed that loss of PPM1H does not alter the overall number of synapses in primary neuron culture [13].

We next asked whether aSyn positioning at the presynapse is affected by PPM1H deficiency. Using high-resolution Airyscan microscopy, we measured the distance between the fluorescence intensity peaks of Bassoon, Homer1, and aSyn in *PPM1H*^+/+^ and *PPM1H*^-/-^ neurons (Fig. 1C–D). The distance between Bassoon and Homer1 was unchanged across genotypes, indicating that synaptic cleft width is not altered by PPM1H loss (Fig. 1E). In contrast, the distance between Bassoon and aSyn was significantly greater in *PPM1H*^-/-^ neurons compared to *PPM1H*^+/+^ controls (0.122 µm vs. 0.084 µm) (Fig. 1F), suggesting that loss of PPM1H shifts aSyn localization away from the presynapse.

To assess whether this effect depends on LRRK2 kinase activity, we next treated *PPM1H*^-/-^ neurons with the LRRK2 kinase inhibitor MLi-2. Consistent with previous work, MLi-2 significantly reduced pT73 RAB phosphorylation in *PPM1H*^-/-^ neurons (Fig. 1G–H) [13]. While MLi-2 treatment did not alter the distance between Bassoon and Homer1, it significantly reduced the distance between Bassoon and aSyn fluorescence intensity peaks (Fig. 1I–L). In DMSO-treated *PPM1H*^-/-^ neurons, the distance between the presynaptic marker and aSyn averaged 0.118 µm. Strikingly, MLi-2 treatment restored this distance to 0.084 µm, matching the average value observed in untreated *PPM1H*^+/+^ neurons (Fig. 1L). These results demonstrate that altered aSyn localization in PPM1H-deficient neurons is dependent on LRRK2 kinase activity and suggest that this effect is mediated by elevated levels of LRRK2-phosphorylated RAB proteins.

The downstream mechanisms by which phospho-RABs affect aSyn positioning at presynaptic terminals remain to be elucidated. One possibility is that altered aSyn positioning reflects impaired local turnover through autophagy, as presynaptic terminals are major sites of autophagosome formation and previous studies have shown that LRRK2-mediated RAB hyperphosphorylation disrupts axonal autophagy [13–15]. Alternatively, altered aSyn positioning may be connected to defects in the trafficking of synaptic vesicle precursors (SVPs) caused by LRRK2-dependent phosphorylation of RAB3 [16]. It may also be linked to the recently reported disorganization of presynaptic release sites, which has been proposed to result from impaired interactions between phospho-RAB3 and the active-zone effectors RIM1 and RIM2 [17].

Since aSyn has been implicated in modulating neurotransmitter release at presynaptic terminals, the altered positioning described here may contribute to previously reported effects of pathogenic LRRK2 on synaptic transmission (reviewed in [18]). However, the precise functional consequences of changes in the spatial relationship between aSyn and Bassoon remain to be determined.

In sum, our results show that loss of PPM1H alters the positioning of endogenous aSyn at presynaptic terminals, increasing its distance from the presynaptic marker Bassoon. Treatment with the LRRK2 kinase inhibitor MLi-2 rescued this effect, strongly indicating that altered localization is mediated by elevated levels of LRRK2-phosphorylated RAB proteins. Together with previous work [12], our findings support a model in which intrasynaptic aSyn localization is sensitive to RAB phosphorylation status, such that reduced RAB phosphorylation decreases the distance to the presynaptic terminal, whereas elevated RAB phosphorylation increases it.

## Electronic supplementary material

Below is the link to the electronic supplementary material.

Supplementary material 1

## Data Availability

All data generated or analyzed during this study are included in this published article and its supplementary information files.

## Abbreviations

aSyn: Alpha-synuclein
DIV: Day in vitro
KO: Knockout
LRRK2: Leucine rich repeat kinase 2
PD: Parkinson disease
PPM1H: Protein phosphatase 1 H
SVP: Synaptic vesicle precursor
WT: Wild-type
