# Supplementary material for: PPM1H loss alters intrasynaptic alpha-synuclein localization in a LRRK2-dependent manner
Source: Mol Brain. 2026 Sep 29;19:66. doi: 10.1186/s13041-026-01352-9 (PMC13621673; doi:10.1186/s13041-026-01352-9)
Supplement: Supplementary file 1 [file 13041_2026_1352_MOESM1_ESM.docx]

**KEY RESOURCES TABLE**

| **REAGENT or RESOURCE** | **SOURCE** | **IDENTIFIER** |
| --- | --- | --- |
| **Antibodies** | | |
| Anti-RAB10 antibody, Mouse Monoclonal | Nanotools | 0680-100/Rab10-605B11,  RRID: AB_2921226 |
| Anti-RAB10 (phospho T73) antibody, Rabbit Monoclonal | Abcam | ab230261,  RRID: AB_2811274 |
| Anti-Rabbit IgG-IRDye 800CW, Donkey Polyclonal | LI-COR Biosciences | 926-32213,  RRID: AB_621848 |
| Anti-Mouse IgG-IRDye 680RD, Donkey Polyclonal | LI-COR Biosciences | 926-68073,  RRID: AB_10954442 |
| Anti-αSynuclein antibody, Mouse Monoclonal | BD | 610787,  RRID: AB_398108 |
| Anti-Bassoon antibody, Guinea Pig Monoclonal | Synaptic Systems | 141318,  RRID: AB_2927388 |
| Anti-Homer1 antibody, Rabbit Polyclonal | Synaptic Systems | 160003  RRID: AB_887730 |
| Anti-MAP2 antibody, Chicken Polyclonal | Abcam | ab5392,  RRID: AB_2138153 |
| Anti-Chicken Alexa Fluor 405, Goat Polyclonal | Thermo Fisher | A-48260  RRID: AB_2890271 |
| Anti-Rabbit Alexa Fluor 488, Goat Polyclonal | Thermo Fisher | A-11034, RRID: AB_2576217 |
| Anti-Mouse Alexa Fluor 546, Goat Polyclonal | Thermo Fisher | A-11030,  RRID: AB_2737024 |
| Anti-Chicken Alexa Fluor 647, Goat Polyclonal | Thermo Fisher | A-21449,  RRID: AB_2535866 |
| **Chemicals, Peptides, and Recombinant Proteins** | | |
| MLi-2 | Tocris | 5756 |
| DMSO | AppliChem | A3672,0100 |
| Poly-L-Lysine (mol wt 70,000 – 150,000) | Sigma-Aldrich | P1274 |
| HBSS (10x) | Thermo Fisher | 14185-052 |
| 1M HEPES | Thermo Fisher | 15630-080 |
| 2.5% Trypsin | Thermo Fisher | 15090-046 |
| Minimum essential medium (MEM) | Thermo Fisher | 11095-072 |
| Horse serum (heat inactivated) | Thermo Fisher | 16050-122 |
| Sodium pyruvate | Thermo Fisher | 11360-039 |
| D-Glucose solution 45% | Sigma-Aldrich | G8769 |
| GlutaMAX | Thermo Fisher | 35050061 |
| B27 Supplement | Thermo Fisher | 17504-044 |
| Neurobasal Medium | Thermo Fisher | 21103-049 |
| Penicillin-Streptomycin | Thermo Fisher | 15140-122 |
| Microcystin-LR | Sigma-Aldrich | 475815 |
| Halt Protease and Phosphatase Inhibitor Cocktail | Thermo Fisher | 78442 |
| EveryBlot Blocking Buffer | BioRad | 12010020 |
| **Critical Commercial Assays** | | |
| BCA Protein Assay Kit | Thermo Fisher | 23225 |
| **Experimental Models: Organisms/Strains** | | |
| Mouse: PPM1H knock-out | Taconic | TF3142 |
| **Software and Algorithms** | | |
| FIJI (Release 2.16.0) | NIH, USA | http://fiji.sc |
| Ilastik (1.4.0rc6) | Berg, Kutra, ilastik Team | https://www.ilastik.org/about.html |
| Prism 10 | GraphPad | https://www.graphpad.com/scientific-software/prism/ |
| RStudio: Integrated Development for R (2022.07.0) | RStudio Team | http://www.rstudio.com/ |
| R package: nlme | Pinheiro J, Bates D, R Core Team | http://CRAN.R-project.org/package=nlme |
| R package: lme4 | Bates D, Mächler M, Bolker B, Walker S | https://cran.r-project.org/web/packages/lme4/index.html |
| Matlab R2020b | MathWorks | https://www.mathworks.com/products/matlab.html |
| VisiView | Visitron | https://www.visitron.de/products/visiviewr-software.html |
| LI-COR Image Studio | LI-COR | https://www.licor.com/bio/image-studio/ |
| Adobe Illustrator 2025 | Adobe | https://www.adobe.com/products/illustrator.html |
| ZEN Blue | Zeiss | https://www.zeiss.com/microscopy/de/produkte/software/zeiss-zen.html |
| **Other** | | |
| 12 mm #1.5 glass coverslips | R. Langenbrinck GmbH | 01-0012/5 |
| ProLong Gold Antifade Mountant | Thermo Fisher | P36930 |

**Primary neuron culture**

*PPM1H* KO mice (Taconic #TF3142) were kindly provided by Dario Alessi, University of Dundee, and have been previously described.^33^ A license for maintaining a breeding colony of *PPM1H* KO mice was obtained from Taconic. All animal experiments were approved by the local animal research council and complied with the legislation for the management and care of laboratory animals in the state of Lower Saxony, Germany.

Coverslips were coated with poly-L-lysine (PLL) prior to dissection. Glass coverslips were placed in a beaker, covered with 0.25% acetic acid, and agitated overnight at 30 rpm. The next day, the acetic acid was removed, and coverslips were washed twice with 100% ethanol and stored in 100% ethanol at 4 °C in a covered beaker. One day before neuronal dissection, ethanol-stored coverslips were air dried, transferred to 24-well plates, and covered with 100 µg/mL PLL solution. After overnight incubation at 37 °C, coverslips were washed twice with ddH_2_O, and attachment medium (MEM supplemented with 10% horse serum, 33 mM D-glucose and 1 mM sodium pyruvate) was added to each well.

Cortical neurons were prepared from littermate *PPM1H^+/+^*, *PPM1H^+/-^*, and *PPM1H^-/-^* embryos of either sex at embryonic day 15.5 (E15.5). Tails were collected for genotyping after embryo decapitation. Cortices were isolated individually, digested with 0.25% trypsin, and mechanically dissociated in attachment medium using a 5 mL serological pipette. Cell viability and density were determined by mixing 10 µL of the cell suspension with 10 µL Trypan Blue and counting using an automated cell counter. Neurons were plated in attachment media at a density of 100,000 cells per well on PLL-coated 12 mm #1.5 coverslips in 24-well plates. After 2 h, attachment medium was replaced with 400 µL of maintenance medium (Neurobasal supplemented with 2% B-27, 33 mM D-glucose, 2 mM GlutaMAX, 100 U/mL penicillin and 100 µg/mL streptomycin) per well. Medium was exchanged twice per week by removing 120 µL and adding 150 µL of fresh maintenance medium per well.

For LRRK2 kinase inhibition experiments, cortical neurons were treated on day 5 in vitro (DIV5) with either 300 nM MLi-2 or the equivalent volume of DMSO (vehicle control). Cultures were fed with maintenance medium containing MLi-2 or DMSO until fixation.

**Immunofluorescence**

DIV21 cortical neurons were fixed using 4% paraformaldehyde (PFA) and 4% sucrose in phosphate-buffered saline (PBS). The fixative was pre-warmed to 37 °C in a water bath. Culture medium was carefully removed from the wells, and 200 µL of fixative was added per coverslip in a 24-well plate. Cells were incubated in the fixative for 9 min at room temperature (RT).

After fixation, coverslips were washed twice with PBS and then blocked and permeabilized for 1.5 h at RT with blocking solution containing 5% normal goat serum (NGS) and 1% bovine serum albumin (BSA) in sterile-filtered PBS supplemented with 0.1% Triton X-100. Next, neurons were incubated overnight at 4 °C with primary antibodies (see Key Resource Table) diluted in blocking solution. The next day, coverslips were washed three times with PBS at RT and subsequently incubated for 1 h at RT in the dark with secondary antibodies diluted in blocking solution. Coverslips were then washed three times with PBS and mounted cell-side down onto microscopic slides using 7 µL ProLong™ Gold antifade reagent. Slides were allowed to cure for 24 h at RT protected from light and were then stored at −20 °C until imaging.

Immunolabeled neurons were imaged using a Zeiss LSM800 confocal microscope equipped with a 63× oil immersion objective. Images were acquired with ZEN software (Zeiss) in Airyscan mode to enhance spatial resolution and signal-to-noise ratio.

**Immunoblotting**

Neurons were washed twice with ice cold PBS and lysed with RIPA buffer (50 mM Tris-HCl, 150 mM NaCl, 0.1% Triton X-100, 0.5% sodium deoxycholate, 0.1% SDS) supplemented with 1x Halt Protease and Phosphatase inhibitor and 2 µg/mL microcystin-LR. Lysates were centrifuged for 10 min at 16,200 g, and protein concentration of the supernatant was determined by BCA assay. Proteins were resolved on 15% SDS-PAGE gels and transferred to Immobilon-FL PVDF membranes (Millipore) using a wet blot transfer system. Membranes were blocked for 5 minutes with EveryBlot blocking buffer and incubated overnight at 4 °C with pT73 RAB10 and total RAB10 primary antibodies (see Key Resource Table). The next day, membranes were washed four times with TBS containing 0.1% Tween-20 (TBS-T) and incubated for 1 h at RT with IRDye-conjugated anti-mouse and anti-rabbit secondary antibodies (LI-COR, see Key Resource Table). After four additional washes in TBS-T, membranes were imaged using an Odyssey CLx Infrared Imaging System (LI-COR). Band intensities were quantified using Image Studio Software (LI-COR).

**Quantification**

Image analysis was performed using Fiji (ImageJ) and ilastik. To quantify the number of aSyn-positive synapses, the machine-learning–based segmentation tool ilastik was trained to generate segmented images for each fluorescence channel. For training, three randomly selected images representing different genotypes were used per marker. Signal regions were manually annotated, while nonspecific background fluorescence was excluded. The trained model was then applied to all genotypes to automatically generate segmented images in an unbiased manner. Segmented images were analyzed in Fiji to quantify aSyn-positive synapses. Triple positive puncta, representing the co-localization of the presynaptic marker Bassoon, the postsynaptic marker Homer1, and aSyn, were identified using the “AND” function of the Image Calculator and quantified using the “Analyze Particles” command.

For analysis of aSyn localization within synapses, fluorescence intensity profiles of Homer1, Bassoon, and aSyn were obtained by line-scan analysis in Fiji. A line was drawn along individual synapses to measure fluorescence profiles of each marker. The distances between fluorescence intensity maxima were quantified to assess aSyn distribution within the synapse.

**Statistical Analysis**

Statistical analyses were performed using RStudio using the *nlme* package. A linear mixed-effects model (LME) was applied, in which genotype or DMSO/MLi-2 treatment was treated as a fixed effect. The experiment/culture and the individual embryo from which neurons were derived were modeled as random nested effects, with embryo nested within experiment. Exact *p*-values are reported in the figure legend. For all quantifications, data from at least three independent experiments (i.e., three independent neuronal cultures derived from different litters) were analyzed.
